# Ogeechee Association

**Published:** 1888-02

**Authors:** 


### OGEECHEE MEDICAL ASSOCIATION.

At a recent meeting of the Ogeechee Medical Association held at Millen, Georgia, the following officers were elected to serve for the year 1888: President, E. W. Lane, of Emanuel county; First Vice-President, J. B. Douglass, of Screven county; Second Vice-President, E. A. Perkins, of Burke county; Third Vice-President, John I. Lane, of Bulloch county; Fourth Vice-President, D. E. Gay, of Emanuel county; Secretary and Treasurer, R. T. Lane, of Emanuel county; Chaplain, Rev. Thos. B. Lanier, of Millen. And at the same time the following members were elected to represent this Association as delegates to the next meeting of the Medical Association of Georgia, to be held in Rome, Ga., on the third Wednesday in April next: G. B. Douglass, of Sylvania, Ga.; J. W. Johnston, of Scarboro, Ga.; E. A. Perkins, of Millen, Ga.

The members of the Ogeechee Association are intelligent and progressive gentlemen of the profession. An abstract of their discussions may be expected to be published occasionally in the RECORD, and will doubtless furnish interesting and instructive matter to our readers.—ED.

